# Learning the Dynamic Treatment Regimes from Medical Registry Data through Deep Q-network

**DOI:** 10.1038/s41598-018-37142-0

**Published:** 2019-02-06

**Authors:** Ning Liu, Ying Liu, Brent Logan, Zhiyuan Xu, Jian Tang, Yanzhi Wang

**Affiliations:** 1 Department of Electrical Engineering and Computer Engineering, Northeastern University, Boston, MA 02115 USA; 2 Division of Biostatistics, Medical College of Wisconsin, Milwaukee, WI 53226 USA; 3 Department of Electrical Engineering and Computer Engineering, Syracuse University, Syracuse, NY 13244 USA; 4 DiDi AI Labs, Beijing, China

## Abstract

This paper presents the deep reinforcement learning (DRL) framework to estimate the optimal Dynamic Treatment Regimes from observational medical data. This framework is more flexible and adaptive for high dimensional action and state spaces than existing reinforcement learning methods to model real-life complexity in heterogeneous disease progression and treatment choices, with the goal of providing doctors and patients the data-driven personalized decision recommendations. The proposed DRL framework comprises (i) a supervised learning step to predict expert actions, and (ii) a deep reinforcement learning step to estimate the long-term value function of Dynamic Treatment Regimes. Both steps depend on deep neural networks. As a key motivational example, we have implemented the proposed framework on a data set from the Center for International Bone Marrow Transplant Research (CIBMTR) registry database, focusing on the sequence of prevention and treatments for acute and chronic graft versus host disease after transplantation. In the experimental results, we have demonstrated promising accuracy in predicting human experts’ decisions, as well as the high expected reward function in the DRL-based dynamic treatment regimes.

## Introduction

Medical treatments often compose a sequence of intervention decisions that are made adaptive to the time-varying clinical status and conditions of a patient, which are coined as *Dynamic Treatment Regimes* (DTRs^1^). “How can we optimize the sequence of specific treatments for specific patients?” is a central question of *precision medicine*. More specifically, the scientific question our paper focuses on is the determination of the optimal DTRs to maximize the long-term clinical outcome.

When the straightforward rule-based treatment guidelines are difficult to be established, the statistical learning method provides a data-driven tool to explore and examine the best strategies. These data driven approaches leverage on the technology advances to collect the increasingly abundant medical data (e.g., clinical assessments, genomic data, electronic health records) from each individual patient to meet the promise of individualized treatment and health care.

The problem of identifying the optimal DTRs that maximize the long-term clinical outcome using *reinforcement learning*^2^ has received much attention in the statistics community^?,^^3–11^. The existing DTR methods are proposed on *Sequential Multiple Assignment Randomized Trial* (SMART)^12^, in which the methods for DTR optimization are limited to clearly defined homogeneous decision stages and low-dimensional action spaces. They are difficult to implement using observational data (such as electronic medical records, registry data), which exhibit a much higher degree of heterogeneity in decision stages among patients, and the treatment options (i.e., the action space) are often high-dimensional. The existing methods can only analyze certain simplification of stage and action spaces among the enormous ways. Simplification by human experts might not lead to the optimal DTRs and in many cases there is no clear way of simplification. In addition, the simplification process needs substantial domain knowledge and labor-intensive data mining and feature engineering processes. For example, Krakow^13^ used Q-learning^9^ in the DTR literature to model a simplified problem of our motivating example. They simplified the problem to only consider one drug (ATG application) at time of transplant for GVHD prophylaxis and 100 day acute GVHD treatment, thereby making it a two-stage problem with two actions at each stage. In the actual action spaces we are directly modeling, the GVHD prophylaxis contains 127 drug combinations (of 14 drugs) and 100 day acute GVHD treatment contains 283 drug combinations (of 18 drugs). And the actions were taken not only at the time of transplant and 100 days. As a result, there is a call for methods to expand DTR methodology from the limited application of SMART studies to broader, flexible, and practical applications using the registry and other observational medical data.

To make reinforcement learning accessible for more general DTR problems using observational datasets, we need a new framework which (i) automatically extracts and organizes the discriminative information from the data, and (ii) can explore high-dimensional action and state spaces and make personalized treatment recommendations. *Deep learning* is a promising new technique to save the labor-intensive feature engineering processes. The effective combination of deep learning (deep neural networks) and reinforcement learning technique, named *Deep Reinforcement Learning* (DRL), is initially invented for intelligent game playing and has later emerged as an effective method to solve complicated control problems with large-scale, high-dimensional state and action spaces^14–19^. We implementated the DRL framework by the *deep Q-network* (DQN), which is a value-based DRL method. The DRL/DQN methods are promising to automatically extract discriminate information among decision stages, patient features, and treatment options. In this work, we incorporate the state-of-the-art DRL/DQN into the DTR methodology and propose a data-driven framework that is scalable and adaptable to optimizing DTR with high-dimensional treatment options, and heterogeneous decision stages.

There are emerging works in the literature for DQN’s implementations on medical problems. Reference^20^ proposed a three-step (GAN + RAE + DQN) framework for automatic dose adaptation to treat lung cancer. There is a training set containing 114 retrospective patients and a testing set of 38 patients. Because of the limitation in the number of patients, the DQN was trained on the simulated dataset where “virtual patients” were generated using the previous two steps GAN and RAE. Besides, the framework is proposed in a special application of the adaptive strategy of radiation dose in cancer treatment. In contrast, our framework is proposed for the national or worldwide patient registry database for any disease, where we use actual patient observation data and experience replay to train the DQN. Preprint^21^ proposed to use dueling double-DQN with a prioritized experience replay to learn Sepsis Treatment, where the motivating data is from the EHR database. This working paper^21^ was brought to our attention by one reviewer. The two teams independently work on DRL implementation on big observation medical database. The distinction between the two paper is that our work aims at a long-term disease treatment and management problem. In comparison, the other team is working on the treatment of an acute condition, i.e. Sepsis. Our work is the first and unique general framework proposed for registry databases with similar structure of the motivating Bone Marrow Transplant registry database, which collect long-term follow-up of each patient national wide, throughout the disease course with standard forms for disease-related patient status and treatments.

To demonstrate the effectiveness of the proposed framework, we implement it using a concrete example: Graft Versus Host Disease (GVHD) prevention and treatment for Leukemia patients who have undergone allogeneic hematopoietic cell transplantation (AHCT). The long-term longitudinal follow-up for almost all US patients and some international patients who have undergone AHCT make the Center for International Blood and Marrow Transplant Research (CIBMTR) registry database an ideal existing data set to explore the capacity of artificial intelligence in medical decision making.

Reference^22^ points out that GVHD is a major complication of AHCT. Once established, GVHD is difficult to treat. It can be prevented by selected methods, but often at the expense of an increased risk of relapse, rejection or delayed immune reconstitution^23,24^. Hence, no optimal or even satisfactory prevention and treatment methods have been defined. Reference^22^ concluded that the difficulty in composing a standard practice guideline is the lack of solid scientific support for a large portion of procedures used in GVHD prevention and treatment, which calls for further systematic studies to compare different strategies. Such clinical needs for methodological innovations in finding the optimal personalized strategies can be largely resolved in the proposed study.

More specifically, in this paper we develop a data-driven DRL framework for the optimal DTR, comprising the prevention and treatment of both acute and chronic GVHDs, as well as the initial conditioning (chemotherapy) after the transplantation. The DRL framework, which deals with heterogeneous decision stages (states) and high-dimensional action space, consists of two steps at each decision stage. The first step is to build a feed-forward deep neural network to predict experts’ treatment with a high-dimensional action space. The second step is to estimate the *value function* of DTRs for strategies composed of the top expert actions with the highest probability from the first step. The state and action spaces, as well as reward function, are carefully selected, and effective dimensionality reduction techniques such as the *low variance filter* are utilized to mitigate the shortcoming of limited data in the database. The similar states have similar encoded representations. In the experimental results, we have demonstrated promising accuracy in predicting human experts’ decisions, as well as the high expected reward function in the DRL-based dynamic treatment regimes.

## Results

In this section, we present the performance of the DRL framework for optimizing the sequence of treatments in prevention and treatment of both acute and chronic GVHDs. We also present the accuracy of the feed-forward deep neural networks (DNNs) in predicting the initial conditioning (chemotherapy) after the transplantation.

Experiments are conducted based on the CIBMTR registry with data of 6,021 patients. The initial conditioning (to prevent relapse) and GVHD prophylaxis (to prevent GVHD) were administered right before the transplant, thus they are considered as action at time *t* = 0; the treatment of acute GVHD takes place at 100 days and 6 months (180 days); the treatment of chronic GVHD takes place at 100 days, 6 months, 1 year (365 days), and 2 years (730 days). We test DTR within 4 years after transplantation because a large portion of patients’ data are missing after that time, and live patients without relapse can be considered to be cured from the disease.

### Results on Predicting Experts’ Treatment using Deep Neural Networks (DNNs)

We first use two supervised DNNs to predict two expert treatment decisions, respectively, at the time of transplant (*t* = 0), which are the initial conditioning (chemotherapy) to prevent relapse and GVHD prophylaxis to prevent GVHD. The inputs of both DNNs contain donor-patient HLA matching information and demographics. The outcome of the two DNNs are the 127 treatment combinations (of 14 treatment options) for GVHD prophylaxis and 145 treatment combinations (of 19 treatment options) for initial conditioning. The treatment options for GVHD prophylaxis include ATG, monoclonal antibody, FK 506, ECP, MMF, KGF, MAB. The treatment options for initial conditioning include busulfan, cyclophosphamide, total body irradiation, fludarabine, thiotepa, melphalan(l-pam), cytarabine, etc.

**Figure 1:**
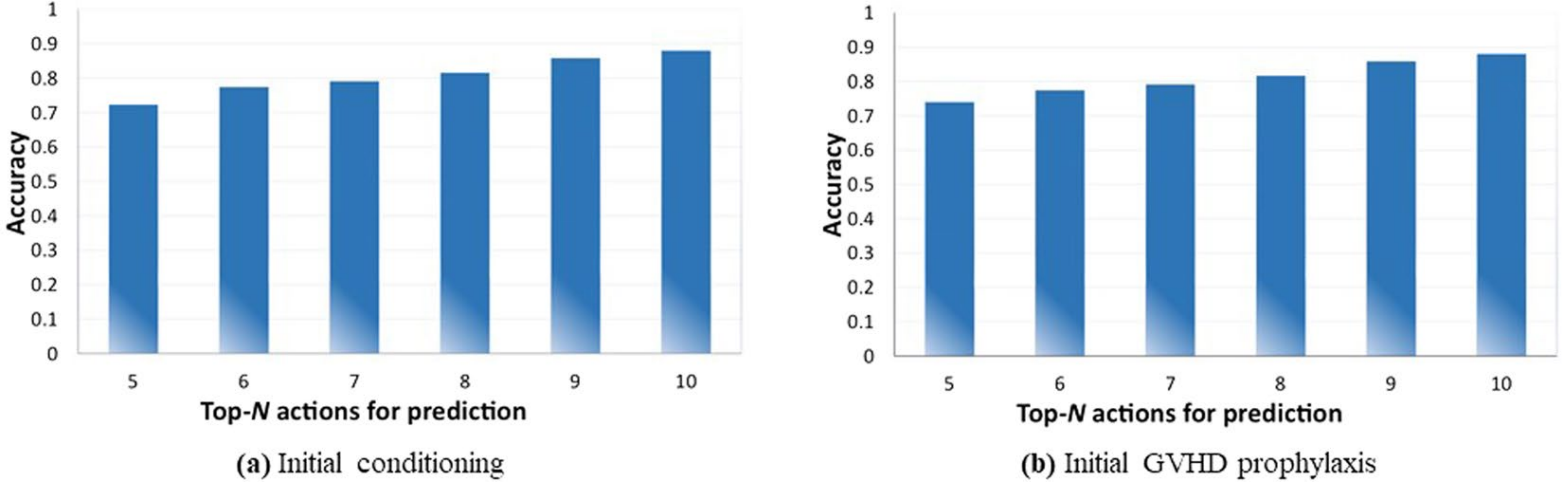
Accuracies on predicting experts’ treatment for initial conditioning and GVHD prophylaxis.

First, we demonstrate in Fig. 1 the prediction accuracies of the initial conditioning and the initial GVHD prevention (prophylaxis). We use 80% of the data set as training data and the remaining 20% for testing data, which is common for deep learning testings. Please note that we utilize the top-*N* prediction accuracy, i.e., the prediction is valid as long as the actual treatment action from human experts is among the top *N* choices suggested by the deep neural network. This top-*N* accuracy is widely utilized for the image recognition such as the ImageNet contest^25^ and other deep learning testings. We can observe that (i) the top-*N* accuracy is in general between 75% and 90%, which shows the effectiveness of the proposed method; and (ii) the top-*N* accuracy will increase with the increase of the *N* value.

**Figure 2:**
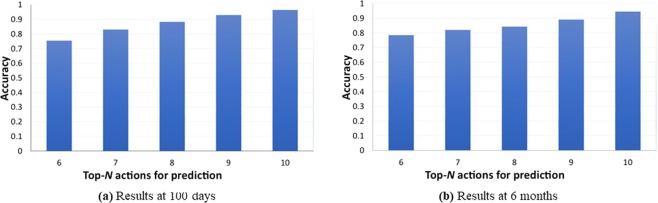
Accuracies on predicting experts’ treatment for acute GVHD.

**Figure 3:**
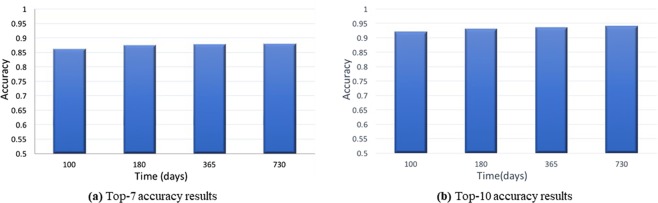
Accuracy results on predicting experts’ treatment for chronic GVHD at 100 days, 6 months, 1 year, and 2 years.

Furthermore, Fig. 2 illustrates the top-*N* prediction accuracy results for acute GVHD treatments at (a) 100 days and (b) 6 months. Figure 3 illustrates the (a) top-7 and (b) top-10 prediction accuracy results for chronic GVHD treatments, at 100 days, 6 months, 1 year, and 2 years. Again we use 80% of the data set as training data and the remaining 20% for testing data. From these two figures, we can derive the following observations. First, the prediction accuracies are in general higher compared with the initial conditioning and GVHD preventions, because the medication for GVHD treatments seems to be more regular compared with the initial treatments. The prediction accuracies are high enough and this shows a first step towards the ultimate goal of DTR using machine intelligence. Next, for the chronic GVHD treatment, the prediction accuracy will increase when time elapses, i.e., the prediction accuracy at 180 days is higher than that at 100 days, and the accuracy at 1 year will be even higher. The reason is that the patients will become more stable and easy for treatment when chronic GVHD occurs or prolongs at a later time.

### Results on DQN-based Value Function Estimation and Making Recommendations

In this section, we provide experimental results on the effectiveness of the DRL-based DTR framework for acute and chronic GVHD treatments, i.e., using DQN for value function estimation and making recommendations. Because of the limited data size to train an overall large DQN model, we build separate DQN models for the treatments of acute and chronic GVHDs. Details about the DQN notations and models are provided in the next section. Again we use 80% of the data set as training data and the remaining 20% for testing data.

The states {*s*_*t*_:*t* = 0, 1, 2, 3, 4} we consider for the DQN at all time points are vectors, which consist of the initial patient features and patient disease status indicators: whether the patient is alive, having acute GVHD, chronic GVHD and relapse. Patients who developed acute GVHD (aGVHD) can later develop chronic GVHD (cGVHD). Therefore, we include cGVHD as a status and derive a reward from this status in the aGVHD network. In the aGVHD network, we also include the cGVHD as a status, because a patient can develop aGVHD before cGVHD. And it can be an important predictor in deciding the cGVHD treatment. This state vector is also used to define the immediate outcome of the previous step in the following way. When *s*_*t*_ belongs to each of the following four categories, reward *r*_*t*−1_ will be defined as follows:

1. *r*_*t*−1_ = 1, if the patient is alive, relapse-free and GVHD-free at time *t*.
2. *r*_*t*−1_ = 0.8, if the patient is alive with acute GVHD at time *t*.
3. *r*_*t*−1_ = 0.7, if the patient is alive with chronic GVHD at time *t*.
4. *r*_*t*−1_ = 0, if the patient is dead at time *t*.

This immediate reward can be viewed as a heuristic quality-of-life measurement. And the cumulative reward, as the sum of these immediate rewards across stages, is also meaningful as quality-of-life measurements.

The action space of DQN for acute GVHD treatment is the 283 drug combinations (from 18 drugs) prescribed at each time step. The action space of DQN for chronic GVHD treatment is the 271 drug combination (from 24 drugs) at each time step. The drugs to treat acute GVHD includes ATG, campath, anti CD25, cyclosporine, ECP, infliximab, MTX, MMF, corticosteroids (systemic), corticosteroids (topical), sirolimus, FK 506, ursodiol, etanercept, thalidomide, etc. The 24 drugs to treat chronic GVHD include some similar drugs with acute GVHD treatment such as ATG, Campath, Anti CD25 and some other drugs such as Infliximab and Lamprene.

To evaluate the performance, we first present the reward comparisons on the testing data set. The results in Table 1 compare the DQN policy, the observed human actions in the test set, random forest and the one-size-fit-all policy that administrates the treatment with the highest frequency to all patients. The reward is computed using the average of all patients who actually follow the recommendation from each policy. Results demonstrate that the mean reward of DQN is better than the other off-the-shelf methods. We also provide the confidence intervals, estimated through 5,000 bootstraps of the testing data. The DQN has a higher mean reward, but the confidence interval is overlapped with the other strategies. This is due to the limited training and testing sample size of the currently available patient cohort. The machine learning methods such as DQN and Random Forest can both provide a data-driven personalized treatment recommendation according to the patients’ features. Compared to Random Forest, the statistical strength of DQN is higher with more training data. In contrast, the one-size-fit-all method suggests all patients have the same treatment without personalization. The expert’s treatment is based on clinical guidelines, doctor’s own experience and knowledge. On one hand, machine learning methods provide the possibility to incorporate information and complex patterns in the database to help make the decision. However, it is still in its early phase for facilitating actual decision making for doctors and patients.

**Table 1:** Reward comparison among the proposed DQN method, one-size-fit-all, random forest, and experts’ treatment.

Treatments	Method	Reward	95% Confidence Interval
AGVHD	DQN	0.717	(0.683, 0.729)
	one-size-fit-all	0.693	(0.659, 0.705)
	Random forest	0.677	(0.666, 0.703)
	experts’ treatment	0.673	(0.663, 0.694)
CGVHD	DQN	0.706	(0.678, 0.722)
	one-size-fit-all	0.684	(0.671, 0.712)
	Random forest	0.672	(0.663, 0.713)
	experts’ treatment	0.671	(0.661, 0.697)

One challenge for using the retrospective dataset to build DQN model is evaluating the DQN performance with the limited number of test data. In existing literature of DQN’s application in medical care problems^20^, DNNs and generative adversary networks (GANs) have been utilized to generate additional virtual training data as well as evaluating the performance. As a result, we propose a second method to evaluate DQN, which is to separately train a DNN to predict rewards, and then compute the expected reward for the top-1 DQN recommendations. In the actual testing data, the top-1 recommendation from DQN is rarely the actual observed treatment, and the DNN will then provide an estimation of the counterfactual reward.

**Figure 4:**
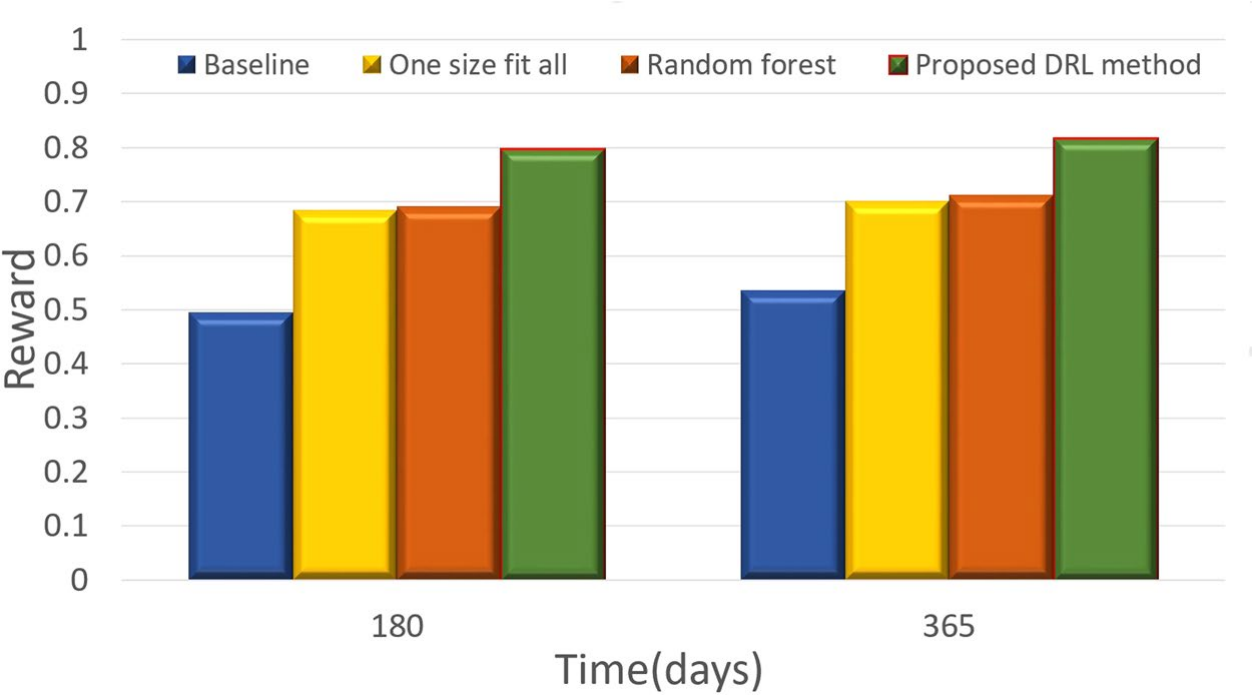
Comparison results between the proposed DRL method and the baseline (details in the context) for acute GVHD treatment.

**Figure 5:**
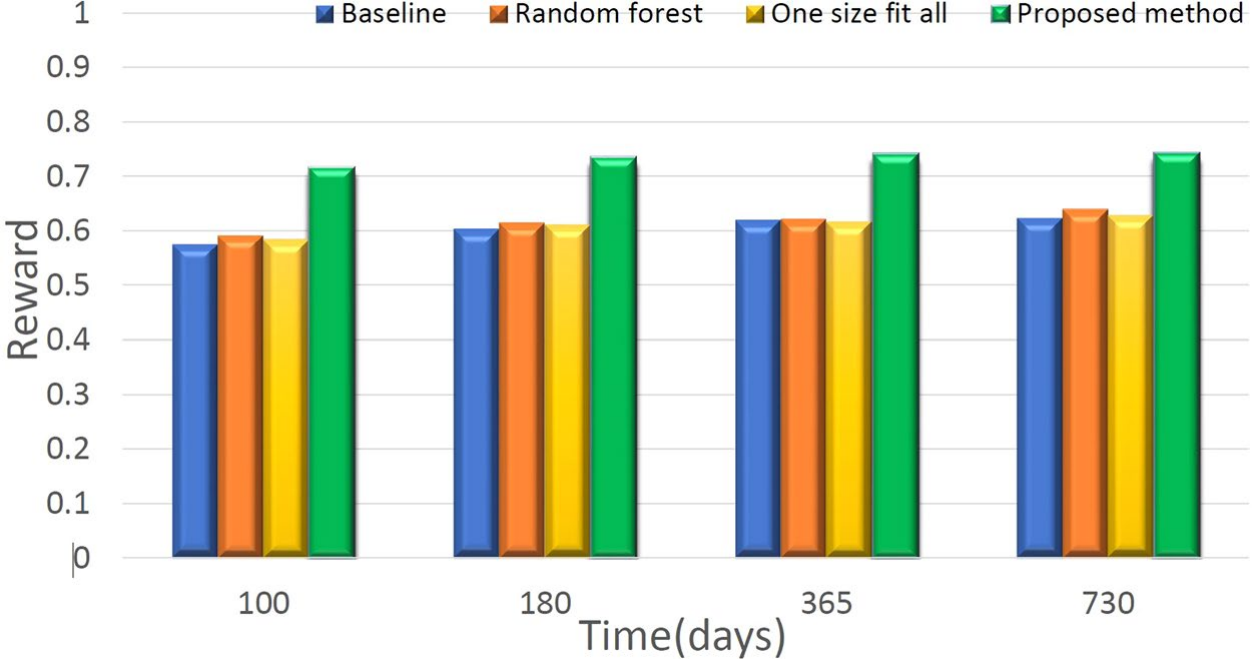
Comparison results between the proposed DRL method and the baseline (details in the context) for chronic GVHD treatment.

Figure 4 illustrates the comparison results between the proposed DQN method and other policies for acute GVHD treatment, while Fig. 5 shows the comparison results for chronic GVHD treatment. All rewards were computed as the predictions from DNN. The DQN and random forest results are based on the top-1 choice of the recommended actions; one-size-fits-all uses the most frequent action and results in corresponding rewards, and the baseline method uses the average of DNN’s estimated rewards for actions excluding DQN’s top recommendation. Despite the limited data, we can still observe that the proposed DQN method outperforms the other methods both for acute and chronic GVHD treatments, which illustrates the effectiveness of using the DRL method for making recommendations in DTR.

## Discussion

In this work, we present a machine learning strategy from an observational dataset to address the decision-making problem of GVHD prevention and treatment. The motivating database has the data structure providing the advantage to implement DRL framework or estimating DTR. It has the nationwide long-term follow-up of each patient throughout the disease course with standard forms. In contrast, it is usually hard, if not impossible, to mine the EHR for long-term patients’ status and treatment decisions. Despite the challenge of identifying meaningful status, treatments and long-term outcomes in the EHR database; one fatal challenge is that current EHR databases are usually regional, the number of patients for one specific disease within the connected EHR databases is very limited for the purpose of learning multiple stage decision rules. The general fitness of the proposed DRL framework with the registry data structure enables enormous potential problems to be addressed in the motivating registry database. Furthermore, it is not hard to transfer to other disease registry databases with the similar data structure. Although EHR databases are the current most prevailing existing data sources, the data structure might evolve in the future together with the emerging analytical tools to meet the potential of AI to better facilitate medical decisions.

In the clinical fields like our motivating example, there are some pressing sequential decision-making questions. For example, in the leukemia field, one other example is to decide whether transplant is a beneficial strategy compared to non-transplant, under what condition or time transplant will become a better option, and adapting these decisions to personal features. Given the constraints on conducting sequential randomized clinical trials on these questions, it is more practical to start from analyzing the observational data at this point. With the improvement of data collection and machine learning techniques in this field, a data-driven decision support system can provide treatment recommendations for doctors based on supervised learning and reinforcement learning. Furthermore, one can adopt the exploration policy in reinforcement learning for the adaptive treatment recommendations, while the decision is made through doctors and patient’s preference.

It is of significant interests to incorporate this machine learned rule to facilitate treatment decision making and how to update the decision rules in an online fashion when new data are collected. There are some current trends in the mobile health field that combines randomized clinical trial with online reinforcement learning through micro-randomized-trials^26^, where the randomization probability can be adopted in an online manner, in analogy to the exploration techniques in reinforcement learning. The applications can be seen in smoking secession, eating disorder management, and blood glucose management for diabetes patients. However, compared with our motivating example in bone marrow transplant, these existing interventions are easier to perform randomization due to the much fewer number of actions, less profound consequences, fewer treatment options and less complicated feature variables. To make our proposed framework more accessible and make on-line training practical, we call for innovations in the data collecting, sharing, and analytical infrastructure. To explore the true capacity of machine learning (including our method) for improving health care, we need a data infrastructure, that can collect data for the whole population in the format friendly to machine learning, can share the data to researchers easily and safely, and can update the data and model in real time.

The Q-learning method has the theoretical guarantee to converge to optimal policy only under the assumptions for Markov decision process. However, Deep Q-learning does not have the theoretical guarantee for convergence to optimal policy even under the Markov decision process because of the sub-optimality of deep neural networks. The disease progression process does not strictly follow Markov process and the state vector we are considering may not fully capture the patients status. However Q-learning and DQN have demonstrated good performance in a lot of application that Markov (memoryless) property does not hold^18,27^. For future work we will remedy this problem with a model without Markov assumption (e.g. RNN), taking the history information into account.

## Methods

In this section we discuss in details the DRL framework for the optimal DTR, comprising the prevention and treatment of both acute and chronic GVHDs, as well as the initial conditioning after transplantation. We first provide a DQN framework which can deal with complicated control problems with high-dimensional state spaces, and then the cohort retrieving and data pre-processing, problem formulation, state and action spaces, the reward function, and optimization techniques of the proposed DRL framework for precision medicine.

### The DQN Framework for Complicated Control Problems

In this section we give a brief introduction of DQN framework and context specific definition of the key components of reinforcement learning, namely the time varying state {*s*_*t*_}, action {*a*_*t*_} and reward {*r*_*t*_}. The goal of reinforcement learning is to optimize the policy *π*, which is a mapping from the state to an action, that would yield the optimal cumulative reward ${R}_{t}={\sum }_{k=t}^{T}\,{\gamma }^{k-t}{r}_{k}$. *r*_*t*_ is the reward at time *t* and *γ* is the discount rate in a discrete-time system. In Q-learning, we define the optimal action-value function

1 $$Q(s,a)=\mathop{{\rm{\max }}}\limits_{\pi }\,E({R}_{t}|{s}_{t}=s,{a}_{t}=a,\pi ).$$

The DQN framework is an extension of conventional reinforcement learning and can be utilized to solve complicated control problems^14–16^. A DNN is utilized to derive the correlation between each state-action pair (*s*, *a*) of the system under control and the corresponding value function *Q*(*s*, *a*).

In order to construct a DNN with a good accuracy, the DNN needs to accumulate enough samples of *Q*(*s*, *a*) value estimates and the corresponding state-action pairs (*s*, *a*). It can be a model-based procedure or obtained from actual measurement data^16^, in which the latter is the case for optimal DTRs in precision medicine. In the original proposed DQN for tasks in robotics or AI for playing games, the procedure includes simulating the control process, and obtaining the state transition profile and estimations for *Q*(*s*, *a*) value, using an arbitrary but gradually refined policy. In the health-care retrospective analysis, the on-policy training is not possible because new data for executing the refining policy cannot be gained on actual patients. Thus we trained our model through experience replay^14^. The state transition profile is stored in an experience memory *D* with capacity *N*_*D*_. According to the inventors of DQN^15^, the use of experience memory can smooth out learning and avoid oscillations or divergence in the parameters. Based on the stored state transition profile and *Q*(*s*, *a*) value estimates, the DNN is constructed with weight set *θ* trained using standard training algorithms such as backpropagation based stochastic gradient descent algorithms. The overall procedure is shown in the first part of Algorithm 1.

**Algorithm 1:**
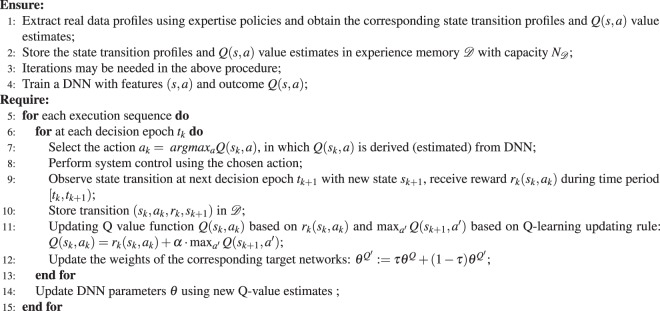
Illustration of DQN Framework.

The deep Q-learning technique is utilized based on the DNN to select actions and update Q-value estimates. More specifically, at each decision epoch *t*_*k*_ of an execution sequence, suppose the system under control is in the state *s*_*k*_. The DQN agent performs inference using DNN to obtain the *Q*(*s*_*k*_, *a*) value estimate for each state-action pair (*s*_*k*_, *a*). The action with the maximum *Q*(*s*_*k*_, *a*) value estimate is selected. After choosing an action denoted by *a*_*k*_, the DQN agent receives total reward *r*_*k*_(*s*_*k*_, *a*_*k*_) during [*t*_*k*_, *t*_*k*+1_) before the next decision epoch *t*_*k*+1_, and this leads to Q-value updates. To mitigate the potential oscillation of the inference results of the DNN^28^, the reference work has proposed to utilize target network which is a duplicate DNN $\hat{Q}$ for Q-value estimate updating. We employ target networks $Q^{\prime} (\cdot )$ to improve learning stability. The target network is a copy of the Q-value network and is used to perform inference of *Q*(*s*_*k*+1_, *a*′). The weights of the target network are updated by slowly tracking of the updated parameters in the Q-value network: $\theta ^{\prime} \leftarrow \tau \theta +(1-\tau )\theta ^{\prime}$ with $\tau \ll 1$. This constraint can significantly improve the stability of learning. The target networks are clones of the original actor or critic networks, whose weights ${\theta }^{Q^{\prime} }$ are initialized in the same way as their original networks but are slowly updated. At the end of the execution sequence, the DNN is updated by the DQN agent using the recently observed Q-value estimates in a mini-batch manner, and will be employed in the next execution sequence. The overall procedure is shown in the second part of Algorithm 1.

As can be observed from the above procedure, the DQN framework is highly scalable for problems with a large state space, which is distinctive from the conventional reinforcement learning techniques. On the other hand, the DQN framework requires an enumerable action space due to the fact that at each decision epoch the DQN agent needs to enumerate all possible actions at the current state and perform inference using the DNN to derive the optimal *Q*(*s*, *a*) value estimate (and corresponding optimal action).

#### Retrieving the Target Cohort and Pre-Processing Data

**Figure 6:**
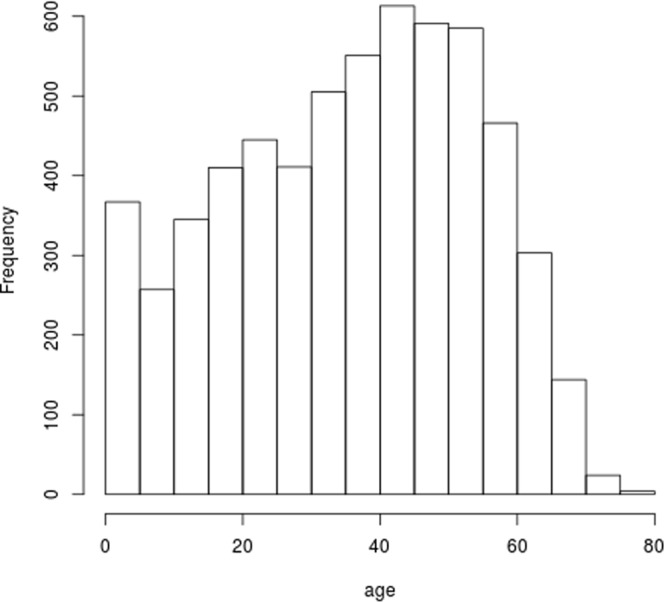
Histogram of Patient Ages in the Data Set of Interests.

In this work, we retrospectively analyzed non-interventional and de-identified existing CIBMTR registry datasets. All patients to be included in the study have already signed informed consent for the data to be used for research. The cohort of patients used for this analysis consists of 6,021 patients diagnosed with Acute Myeloid Leukemia (AML) who have undergone HCT between 1995 and 2007. Due to the discrete data collection scheme, we have higher quality data on the onsets of GVHD conditions and the subsequent treatment decisions in a discrete-time frame indicating the occurrence between two follow-up times. The exact date and sequence of treatment decisions between two periods of time are missing or not recorded to a greater extent. In this work, the state and action are considered to be the state and action taken at the time each form was recorded. We consider relapse and death as terminal states and occurrences of acute or chronic GVHD as transient states. We consider baseline features of patients and donors that have been shown to affect GVHD and survival rates in clinical studies, including patient’s age, gender, and co-morbidity information (including diabetes, seizure, hypertension, etc.). It also includes important patient and donor relationship to patient and matching information, as well as donor’s gender. This cohort includes both pediatric and adult patients. We include the histogram of age in Fig. 6. And the Human Leukocyte Antigen (HLA) matching results of patients are presented in Table 2.

**Table 2:** Matching Information of Patients and Donors in the Data Set of Interests.

Identical Sibling	Other Relative	URD Well Matched	URD Partially Matched	URD Mismatched	Other
3877	451	686	433	173	401

### Details of Deployment of the DRL Framework on the Data Set

**Figure 7:**
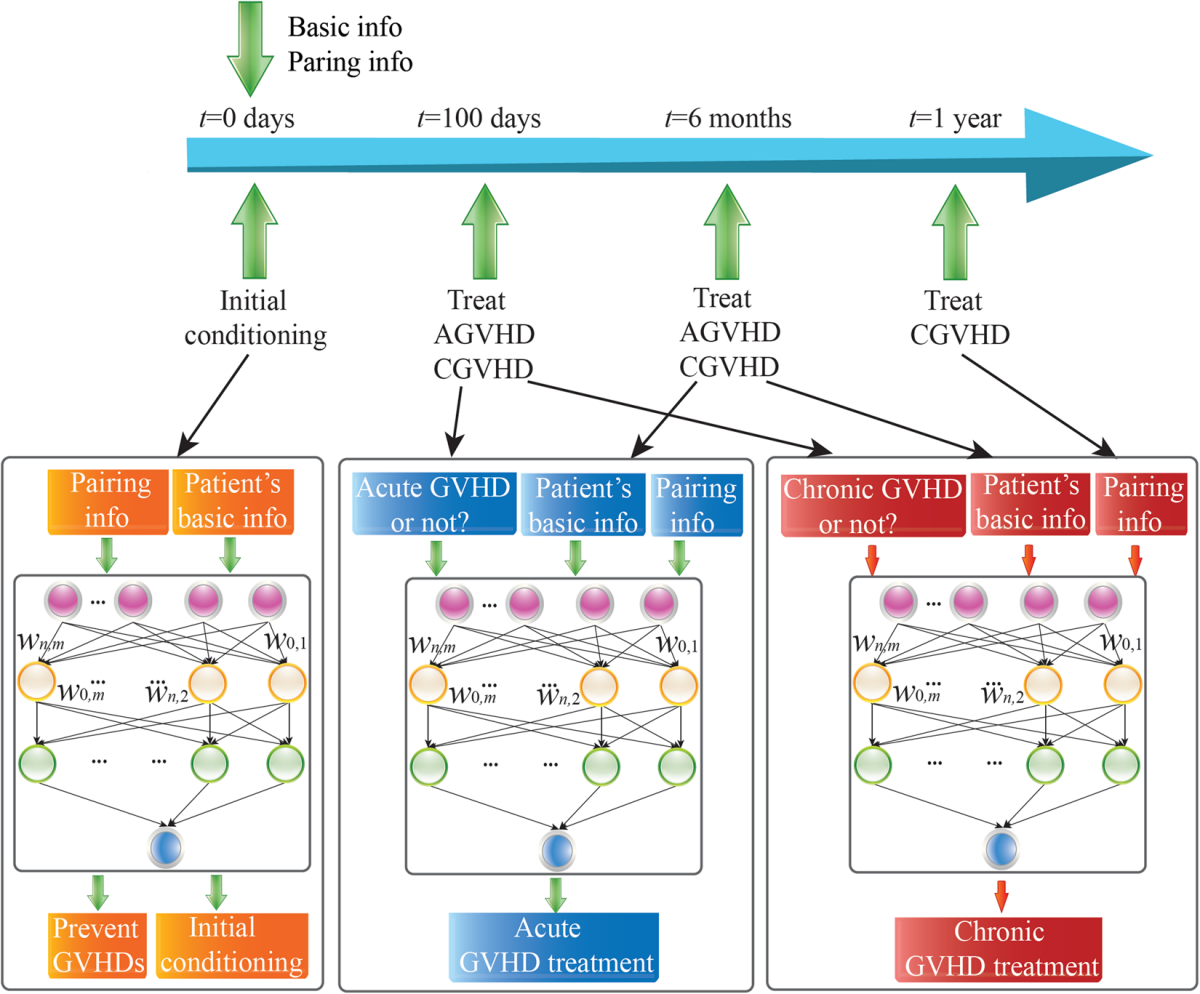
The proposed DRL/DQN framework for prevention and treatment of GVHD, as well as initial conditioning.

We present the time frame and their corresponding information and actions in Fig. 7. We build separate models for decision recommendation for cGVHD and aGVHD. Our proposed framework is realized in a DQN and a screening DNN, where the screening DNN is to predict expert actions. Among the DQN recommendations, we only consider the most likely actions of experts. The variability is high in the estimated value function from DQN for the actions that never appeared or only appear in a couple of times. These actions correspond to the treatments that experts have a small probability to assign for patients given their features. Such actions cannot be suggested by the system for reliability reasons and will be screened out by the screening DNN. The details of the above mentioned DQNs and screening DNNs are provided as follows.

First, two DQNs are adopted to make recommendations for acute and chronic GVHD treatments. A four-layer fully-connected neural network architecture is adopted in the DRL/DQN network for acute and chronic GVHD treatments. It consists of the input layer, two hidden layers, and the output layer. The input dimensions for treating both acute and chronic GVHDs are 8. The hidden layers are 32,64. The output dimensions for treating acute and chronic GVHDs are 283 and 271, respectively. The input features (the state space) include the basic information of patients and the HLA matching information. And the output is the action’s Q value. These two DQNs are trained by our DQN algorithm. The learning rate *η* is set to be 10^−3^. In the DQN, we use a replay buffer to store the dataset^28^. The replay buffer is a finite sized cache which can store the sampled transition tuples (*s*_*t*_, *a*_*t*_, *r*_*t*_, *s*_*t*+1_), and it discards the oldest samples when it is full. The replay buffer allows the algorithm to benefit from learning across a set of uncorrelated transitions. Direct implementation of deep Q-learning may cause the network to be unstable during the training process. As a result, we adopt the target network introduced in reference^28^. The target network is a copy of the Q-value network and is used to perform inference of *Q*(*s*_*k*+1_, *a*′). The weights of the target network are updated by slowly tracking of the updated parameters in the Q-value network: *θ*′ ← *τθ* + (1 − *τ*)*θ*′ with $\tau \ll 1$. This constraint can significantly improve the stability of learning. The target network updating parameter *τ* is set as 0.01, and the discount rate of reward *γ* is set as 0.99. The size of replay buffer is 20000.

Second, two screening DNNs are used to select top expert actions with the highest probability from the DQN result. The architecture of these two DNNs are similar to the DQN, which are 8− > 16− > 32− > 283/271 for acute and chronic GVHDs respectively. The input features (the state space) include the union of the basic information of patients (e.g., age, gender, and cormorbilities, etc.) and the HLA matching information between the patient and the donor. And the output is the actual combination of medicines used by the expert.

The whole process of making recommendations are 2 steps. First, the DQN generate the action. Second, the action is selected by the screening DNN, which only consider the highest probability from the DQN result.

In addition, for the initial treatment before the transplantation, the input features (the state space) include the union of the basic information of patients (e.g., age, gender, and cormorbilities, etc.) and the HLA matching information between the patient and the donor. The output label (action) is the combination of medicines to be utilized for the initial treatment which includes the initial conditioning to avoid disease relapse and the GVHD prophylaxis to prevent GVHD. In this step, we adopt the multiple layers, fully-connected neural network as our DNN. The network architecture consists of four layers: the input layer, two hidden layers, and the output layer. The dimension of the input layer is 9 and the two hidden layers have 16 and 32 neurons, respectively. The output dimension is 145 for initial conditioning and 127 for GVHD prophylaxis. We use the Adam Optimizer to train the network, and the learning rate *η* is set to be 10^−4^ ^29^.

## Data Availability

The authors are willing to share the cleaned data and code with the Editorial Board Members and referees upon request. Additional steps of IRB approval maybe needed in accordance with CIBMTR registry policy.
